# Problems of Teaching the Behaviorist Perspective in the Cognitive Revolution

**DOI:** 10.3390/bs3010055

**Published:** 2013-01-08

**Authors:** Charles I. Abramson

**Affiliations:** Laboratory of Comparative Psychology and Behavioral Biology, Departments of Psychology and Zoology, Oklahoma State University, 116 North Murray, Stillwater, OK 74078, USA; E-Mail: charles.abramson@okstate.edu; Tel.: +1-405-744-7492; Fax: +1-405-744-8067

**Keywords:** behaviorism, cognitivism, teaching, textbooks, neobehaviorism

## Abstract

This article offers some personal reflections on the difficulty of teaching the behaviorist perspective in the psychology classroom. The problems focus on the inadequacy of introductory textbooks—which mischaracterize behaviorism, only present the most extreme behaviorist positions, make no mention of the neobehaviorist perspective, fail to discuss that there is no accepted criteria for determining what type of behavior is cognitive, and provide a definition of cognition that is, not only inconsistent across texts, but so broad as to overshadow the behaviorist contributions. Suggestions are provided for instructors on how to present to their students an accurate portrayal of behaviorism.

## 1. Introduction

What is cognition? A look through any introductory textbook and most cognitive texts gives the student an impression that cognition is practically all of psychology. They will see sections on, for example, Cultural Cognition, Analytical Cognition, Holistic Cognition, Neonatal Cognition, Cognition in the Mini-Brain, Cognitive Architecture, and one of my personal favorites, Unconscious Cognition. 

This article offers some personal reflections on the problems associated with teaching principles of behaviorism within the cognitive revolution. I hope to lend a voice to educators such as myself who are dissatisfied, and perhaps even saddened, by a revolution that neglects some of the greatest contributors to the analysis of behavior; by a revolution that misrepresents the behaviorist position in textbooks; by a revolution where traditional behavioral issues are being tossed aside and all but forgotten by a new generation of students [1,2]. It is this dissatisfaction that has led to the publication of this special issue on “What is Cognition?”

I shall briefly comment on several issues that have concerned me as a teacher of psychology. Students are never told, for example, about the wide variety of behaviorist positions, are presented with definitions of cognition that are so broad that they are meaningless at best, and at worst, overshadow the behaviorist contribution to psychology, and are not told that there are no general criteria to determine whether a process is cognitive. The issues voiced in this paper are not unique to me. They have been expressed by many individuals including Frederick Adams [3], Abraham Amsel [4], Howard Cromwell [5], James Grice [6], Vickie Lee [7], Jay Moore [8,9], Geir Overskeid [10], Jaak Panksepp [5] and Thom Verhave [11,12].

To provide the reader with a context for my comments I teach a course on the psychology of learning in a department where I am probably the only behaviorist—at least the only one proud to say so. In my 18 years of teaching both the undergraduate and graduate courses on learning, I am often shocked by how little colleagues and students know about behaviorism apart from the catch-phrases and stereotypes associated with attacks on John B. Watson and B. F. Skinner. Behaviorists are often considered by colleagues as out of touch, anti-intellectual, old fashioned, and one of my personal favorites—simple minded. The cognitivists, on the other hand, are cutting edge, forward thinking, insightful, and entering new frontiers. 

Over the course of a semester, my students are surprised to learn that the behaviorist approach is still vital and has much to recommend it as a scientific enterprise. They are surprised that the behaviorist perspective can provide a framework to study complex human behavior; they are surprised to learn that the behaviorist perspective is more than rats in mazes and pigeons pecking disks, and they become disillusioned with a psychology that fails to teach them viable alternatives to the prevailing cognitive zeitgeist.

The issues, accompanying citations, and teaching exercises presented in this article have been useful as a basis for a dialog in both my undergraduate and graduate courses when behaviorism and cognitivism are discussed. This article will also be of some value to readers of this special issue who may begin to see behaviorism in a more positive light and lead them to a more accurate portrayal of behaviorism in their own classroom environments.

## 2. What Behaviorism are We Talking about?

When discussing behaviorism in the classroom, students (and faculty) are often surprised that there are several different types of behaviorism. Students must learn that when a professor attacks behaviorism they must ask the professor at least three questions: (1) “What form of behaviorism are you talking about?” (2) “If behaviorists focus on observable behaviors what do cognitivists focus on—unobservable behavior?” and (3) “If behaviorists do not reference mental processes, how do you explain the contributions of Hull, Tolman, and Miller and their use of intervening variables?” 

No serious social scientist questions the inaccuracy and racism of lumping Mexicans, Spaniards, and Puerto Ricans into the general category of “Hispanic” or Arapahos, Choctaws, Poncas, or Pawnees into the general category of “Native Americans.” The use of such categories precludes serious comparative analysis, prohibits an understanding of nuances among differing theoretical positions, and leads to the grossest forms of generalization. Yet these same social scientists feel free to lump together the various behaviorist perspectives. Behaviorism has never been a unitary psychological perspective and proponents differ significantly in terms of methodology and theoretical outlook [8,9]. In introductory textbooks, and textbooks devoted to cognition, typically only two types of behaviorism are mentioned those of John B. Watson and B. F. Skinner. 

I would encourage the reader to examine *Behaviorism: A battle line* [13] and to compare its outlook toward behaviorism with their own. This book is noteworthy for several reasons. First, unlike the vast majority of contemporary introductory and cognitive texts, it clearly acknowledges the existence of several different types of behaviorism. In addition to the behaviorism of Watson, there is the behaviorism associated with, for example, John Dewey, Walter B. Pillsbury, Edward L. Thorndike, Edward C. Tolman, Howard C. Warren, and Robert M. Yerkes [14]. Readers interested in offering their students some of the history associated with very early forms of behaviorism should assign Roback [15] and Verhave [11]. Verhave’s work is especially interesting because it highlights the contribution of a little known American Professor of Physiology Joseph R. Buchanan. Buchanan’s book *The philosophy of human nature* [16] contains several laws of association that found their way into formal behaviorist approaches. Students will also benefit on reading some of the early philosophical contributions to behaviorism by, for example, Gottfried W. Leibniz who was not as anti-associationist as many believe [12], Plato [17], and Francis Hutcheson [18]. 

Second, it is interesting to note that all the contributors in *Behaviorism: A battle line* warns that behaviorism as taught in universities and across the United States is a dangerous enterprise and must be stopped. McDougall [19] relates a story describing the reaction of a teacher to the spread of behaviorism in the classroom as “…wherever he goes, he finds Behaviorism rampant in the schools, and that, because he cannot accept it, he finds himself regarded by his colleagues as hopelessly out-of-date” ([19], p. 48). My the times have certainly changed! It is now unchecked cognitivism that is rampaging through our universities and colleges and producing a class of students who know next to nothing about a still vital and vibrant conception of psychology. 

Third, there is the vilification of behaviorism. Each chapter of *Behaviorism: A battle line* is full of malicious comments directed at Watson in particular and behaviorism in general. Many of these comments have a modern ring to them that I am sure the reader will recognize. These comments were ridiculous then as they are over 80 years later. Behaviorism is called a cult, absurd, nonsense, grim, unethical, and poison. It is suggested that an acceptance of behaviorism increases anti-social and criminal behavior, that behaviorism leads to moral decay and, is at the same time a religious cult yet anti-religious, amoral and suppresses artistic expression. This tone is very similar to how democrats portray republicans. As Coffin [20] wrote “So Behaviorism appears as a pathetic figure circling around in the backwash of the widening swiftly flowing stream of science.” ([20], p. 255). For those readers interested in another entertaining early book critical of behaviorism see *The Religion Called Behaviorism* [21]. 

Given such criticism it is remarkable that behaviorism became the dominant form of psychology in the United States for several decades. It is also remarkable that those few still working within the behaviorist perspective continue to make substantial contributions way beyond the small number of contemporary practitioners. To paraphrase Winston Churchill: Never in the field of social science was so much owed by so many to so few.

That various types of behaviorisms exist is an important point often overlooked in the classroom and in textbooks. When professors discuss behaviorism in the classroom they must inform their students that there are several different perspectives just as there are different perspectives to cognitive psychology such as information processing. This is not done. As Amsel [4] aptly points out, usually the only behaviorist positions students are exposed to are those of Watson and Skinner. Even here, when discussing their views, textbook authors focus on the extreme positions. For example, Watson’s early work [22,23] is very different in perspective from his later position after he was forced to leave academia [24]. 

When considering Watson’s extreme positions, authors often mischaracterize it. Consider just a few of the many examples that can be found in *Behaviorism: A battle line* many of which students and faculty believe and repeat to the present day. 

1. Some texts claim that Watson was “…prepared to produce from any human infants given over wholly to his tender mercies a corresponding number of human beings of any desired type, geniuses of the first water, mathematicians, musicians, artists, scientists, statesmen, executives, anything, in fact (other than theologians or metaphysicians), according to specifications given.” ([18], p. 47). 

This statement borders on the outrages and is often used to discredit the entire behaviorist approach. Watson’s full quote on which McDougall’s (see also [25], p. 294) is based, contains several lines that are typically and conveniently left out. These lines are: “I am going beyond my facts and I admit it, but so have the advocates of the contrary, and they have been doing it for thousands of years” ([24], p. 104). When this line is included, Watson’s meaning becomes clear. 

2. “Since such states or attitudes as love, hate, fear, courage, pain, hope, loyalty, and aspiration, cannot so be recorded, they are regarded by the Behaviorist as of no consequence.” ([26], p. 63).

This is not true of Watson (see [27]—“A schematic outline of the emotions”) and it is certainly not true of the group of behaviorists known as neobehaviorists. As but three examples of literally 100s that I can select from, consider the work of O. H. Mower on fear and hope [28,29], Amsel’s work on frustration [30] and Neil Miller’s work on conflict [31]. 

3. The type of behavior that Watson studied is characterized as “Muscular reactions and glandular secretions” ([32], p. 90).

4. “Extreme Behaviorism denies all mental life, including conscious, purposive experience…” ([33], p. 213). 

5. Heredity unquestionably plays a role in our physical and mental make-up ([34], p. 279). 

6. “All human behavior is a matter of stimulus and response” ([25], p. 294).

Even the most causal reader of the original source material by Watson knows that statements 3, 4, 5, and 6 are demonstrably false as characterized by cognitivists. It is vitally important for students to understand the time period and the state of psychology during Watson’s era. Watson [22,23] advocated observation, verbal reports, psychological tests, statistical training, laboratory training, acknowledges the importance of emotions (specifically commenting on fear, rage, love), instinctive responses, and the importance of heredity. In the opening chapter to his *Psychology from the Standpoint of a Behaviorist *[23] he suggested that the training of psychology students include the study of physiology, chemistry, and zoology (read Chapter 1—Problems and Scope of Psychology, especially the section on Preparation for Psychology). 

As Cohen notes [35], Watson’s perspective is characterized by an attempt to catalog behavior, to make observations under laboratory and field conditions, to study developmental influences, to conduct controlled and repeatable experiments in an attempt to understand human nature. He was one of the first to study development, human sexual behavior, behavior modification, and imprinting. This is a behaviorism not of the “glandular squint” as portrayed in textbooks and by cognitivists but a dynamic approach that has impacted many fields including behavior therapy and industry. It has earned the right to be properly discussed in textbooks used to train the next generation of psychology professionals. 

The portrayal of Skinner’s version of behaviorism (known sometimes as radical behaviorism) is also given “short shrift” in textbooks and classroom discussions. Perhaps the most entertaining example of this can be found in a collection of his seminal papers with commentary [36]. What is unique about this volume of collected papers is that he is given the opportunity to respond to the commentaries. His commentaries on the commentaries are interesting because he spends a large portion of his time correcting the inaccuracies the commentators have on his positions. It is well worth reading and incorporating his comments into student reading lists. Moore [8] also describes errors in communicating Skinner’s view of cognitive or mental events. 

In between the so-called extremes of Watson and Skinner’s approaches to behaviorism is an entire group of behaviorists that are shamefully neglected in introductory and cognitive texts. This type of behaviorism is known as neobehaviorism. Neobehaviorism is an approach to theorizing arguably begun by Clark Hull that makes extensive use of intervening variables. The Hullian approach is also known as molecular behaviorism in contrast to the molar behaviorism of Tolman, the contiguity approach of Edwin R. Guthrie, and the radical behaviorism approach of Skinner. All the various behaviorist approaches (even the behaviorism of the “Watsonian Type”) regularly consider what are now called cognitive processes [8]. 

It is important to note that not all neobehaviorists would feel comfortable being labeled a behaviorist. In his chapter on “Behavioristics”, Edwin G. Boring [37] discusses that Clark Hull and his collaborators would be “puzzled if called behaviorists.” Nevertheless, categories are important and the work of Hull and his colleagues clearly fall into the general category of behaviorism and neobehaviorism in particular. 

The neobehaviorists represent some of the most significant figures in the history of psychology and I dare say that few readers of this article have ever heard of them, or their contributions, other than in the context of an historical curiosity. In addition to Hull, Abram Amsel, Neal E. Miller, O. H. Mower, and Kenneth W. Spence, for example, have made many contributions in areas that are now co-opted by cognitivists, many of whom apparently do not even know the history of their own research area. If the reader would like to amuse him or herself during the evaluation of a job candidate’s seminar, simply ask the candidate to describe the behaviorist contribution to the research area that they are supposedly experts on. The response will most often be “never heard of any contribution” and many of your colleagues will think you just asked a trick question. 

Even a shallow look at the *Psychological Review* papers of Clark Hull reveals a real concern with tackling issues such as “Knowledge and purpose as habit mechanisms”, Goal attraction and directing ideas conceived as habit phenomena”, “The mechanisms of the assembly of behavior segments in novel combinations suitable for problem solution”, “Mind, mechanism, and adaptive behavior”, “The problem of intervening variables in molar behavior theory.” These and other topics related to Hull’s *Psychological Review* papers are conveniently collected with commentary in the edited volume of Amsel and Rashotte [38]. At least some of these papers and their commentaries should be assigned to students (and mentioned in introductory and cognitive texts) if students are really to be given a legitimate opportunity to understand what the behaviorist approach has to offer the cognitive one. Webster and Coleman [39] offer some insights why the influence of Hull’s theory declined. 

Hull is certainly not alone in investigating issues that are considered cognitive. The psychological literature from the 1920s through the 1960s literally overflows with behaviorists tacking problems now thought to have originated with contemporary cognitivists. One nice example was reported by the “Connectionist Behaviorist” E. L. Thorndike on learning without awareness (known now as “unconscious cognition!”) [40]. His volume of collected papers is still will worth a look [41]. 

Other examples include the “Contiguous Behaviorist” E. R. Guthrie’s [42] *The psychology of human conflict* (for some interesting extensions of Gurthrie see [43] and the work of Haraway [44,45,46,47], the “Purposive Behaviorist” E. C. Tolman’s *Purposive behavior in animals and men* [48] and see his *Collected papers in psychology* [49]. 

The neobehaviorist Neal E. Miller’s work with John Dollard on the application of behaviorist principles to Freudian theory [50] is especially exciting and worth reading. Miller’s efforts represent a fine example of the vitality and scope of behaviorism—a behaviorism that students never experience. A glance through his volume of collected papers [51] reveals to the student richness in subject area and methodology that they never thought possible for a psychological perspective that is considered “absurd, nonsense, grim, unethical, and poison.” Miller’s collected papers are full of interesting experiments on what is now considered cognitive topics—all of them conducted within a behaviorist perspective. His experiments include work on “Theory and experiment relating psychoanalytic displacement to stimulus-response generalization”, “Learning resistance to pain and fear: effects of overlearning, exposure, and rewarded exposure in context”, and “Failure to find a learned drive based on hunger; evidence for learning motivated by “exploration” [51].

Another example of the behaviorist interest in complex human processes is in the seldom cited work of Arthur W. and Carolyn K. Staats. Staats and Staats [52] cogently demonstrate the richness and vitality of applying the behaviorist approach to complex human behavior. They examine a host of what are now considered cognitive topics. These topics include child development, personality, language, and motivation. Of course, they are not the only behaviorists who attempt to tackle the intricacies of human behavior and are part of the tradition of Watson, Hull, Miller, Tolman, Guthrie, Mower, and Skinner among others. 

Attempts at reconciliation of the cognitive and behaviorist positions are also not mentioned in textbooks. The positions are portrayed as one having replaced the other. This is unfortunate because it further suggests to students that the behaviorist position is outdated and has little to recommend it. A paper by Denny [53] is especially useful in this regard. Denny shows that by modifying the definitions of stimulus and response, cognitive and behaviorist approaches can be reconciled. This attempt is similar to the efforts of MacCorquodale and Meehl [54] that endeavored to reconcile Hull’s theory with the cognitive behaviorism of Tolman. In doing so, they revealed many points of agreement. Miller [55] has also shown that modifying some neobehaviorist concepts can help psychologists better understand motivation and conflict. These papers should be assigned to students to get them to think critically about how the behaviorist and cognitivist perspectives can be combined. 

In addition to presenting the view that the cognitivist position has supplanted the behaviorist position without mentioning attempts to reconcile the two perspectives, textbooks for introductory or cognitive psychology have never in my experience given the student the sense of the excitement and discovery associated with the efforts of behaviorists. The period from the 1920s through the early 1960s is one of the most exciting times in the history of behaviorism, indeed in the history of psychology. This time period is characterized by laboratories working to replicate and extend findings, developing new experimental designs in the area of, for example, latent learning, successive negative contrast, and avoidance learning, creating new apparatus and techniques, and testing the limits of differing conceptualizations of animal and human conduct. I am sure that I am not voicing the popular opinion but it is a real intellectual tragedy, and I would further say intellectually dishonest, that students are not exposed to an accurate account of the behaviorist perspectives in introductory and cognitive classes. This work will never be brought to the attention of a new generation of students if their own faculty do not know of its existence and journals refuse to allow authors to cite the relevant historical literature. 

## 3. Definitions of Cognition in Textbooks

In addition to problems faced by professors who must battle inaccurate and often outrageous portrayals of the behaviorist perspectives, is the definition of cognition. The definition of cognition in textbooks is an important issue for those of use who are behaviorists. Cognition definitions are so broad that they seemly cover every aspect of psychology even those areas that were traditional first developed and stimulated by behaviorists. 

Students rely on textbooks as one of the most important sources of information and the glossary, in particular, helps identify and highlight important terms that the author considers important [2,56,57]. I urge the reader to visit your bookshelves and look at the glossary of your introductory psychology or cognitive textbooks plus the preliminary comments related to the definition of cognition and the behaviorist approach. What you will find are definitions of cognition that cover the entire spectrum of psychology and therefore are essentially meaningless while the definitions of the behavioral perspective are consistent although sometimes wrong when they exclude **“**inner events.” As another exercise, use the thesaurus function on your word professor. If it is like mine, there are various entries for the word “behavior” such as performance, deeds, and actions. If you type in “cognition” there are no entries. 

There is a real need to offer a universally accepted definition of cognition that can be compared to other perspective approaches to psychology. Without a precise definition of cognition, or at least the cognitive perspective, students are left with the impression that there are no serious alternatives to the cognitive model. Those readers, who teach psychology courses from the behaviorist perspective like me, find it difficult to provide students with materials that adequately and fairly present alternative perspectives. This is a serious issue because it affects the training of the next generation of students. 

To document the inconsistencies in definitions of cognition, I took the opportunity to examine eight recent introductory psychology texts. What I found confirms the lack of consistency in the definition of cognition. In contrast to definitions of behaviorism, there is no consensus on what cognitive psychology is and the definitions are designed to cover almost every area of psychology. This is in contrast to definitions of behaviorism that all stress the focus on observables. None of the definitions of cognition mention that a cognitive psychologist does not see a “cognition” or “cognit” they, like the behaviorists only see observables. 

The lack of consistency in cognitive definitions is not a surprise given the history of the field. In what is erroneously considered the first textbook in cognitive psychology (see T.V. Moore’s Cognitive Psychology, [58]; Knapp, [59]). Neisser [60] defines cognitive psychology as “all processes by which the sensory input is transformed, reduced, elaborated, stored, recovered, and used.” Moreover, as Amsel [4] noted, the founding editor of the journal *Cognitive Psychology* when asked to define this field replied that it is “What I like.” At best such a reply precludes any meaningful discussion on what is and what is not cognition and worse disrespects alternative approaches and leads to an influx of such terms as cultural cognition, analytical cognition, holistic cognitive, neonatal cognition, and cognition in the mini-brain.

Ciccarelli and White [61] do not define cognition in the glossary but they do define, cognitive dissonance, cognitive arousal theory, cognitive-behavior therapy, cognitive-meditational theory, cognitive neuroscience, cognitive psychologists, cognitive therapy and cognitive universalism. Behaviorism is defined as “The science of behavior that focuses on observable behavior only.” There is no mention of the existence of various behaviorist perspectives such as neobehaviorism, nor are the problems we investigate such as learning and problem solving mentioned. Yet in the preliminary comments, the cognitive perspective is defined as “Modern perspective that focuses on memory, intelligence, perception, problem solving and learning.” The reader can only assume that by using the word “modern” the authors of the text believe that the behaviorist approach is antiquated. 

Gray [62] also does not define cognition in the glossary but defines cognitive-behavior therapy, cognitive dissonance, and cognitive therapy. Behaviorism is defined, but the definition includes the statement that …”behavior should be understood in terms of its relationship to observable events in the environment rather than in terms of hypothetical events within the individual.” Given my earlier comments on the various types of behaviorisms I hope the reader is aware how uniformed this statement is. When you examine introductory texts for their treatment of behaviorism, you will see that they are wrong to characterize behaviorism this way without mentioning that there are several behaviorist approaches. This statement may or may not be true of the radical behaviorism advocated by B. F. Skinner but it is certainly not true of the neobehaviorists such as Hull and Tolman. In the preliminary comments, cognition is defined as: “The term cognition refers to information in the mind—that is, to information that is somehow stored and activated by the workings of the brain.” The definition of cognition offered by Gray is different than that offered by Ciccarelli and White [61].

Yet a third definition of cognition is presented by Huffman [63]. In the glossary, she defines cognition as “Mental activities involved in acquiring, storing, retrieving, and using knowledge.” Definitions are offered for cognitive behavior therapy, cognitive dissonance, cognitive map, cognitive perspective, cognitive restructuring, cognitive-social theory, cognitive therapy. Behaviorism is not defined. Astonishingly, Clark Hull is listed in a table entry (Table 1.2, page 15) as representing the cognitive perspective! This is simply ridiculous. One would have thought that Tolman would have been a better choice. For those readers who have never heard of Hull or Tolman—both were neobehaviorists. 

A fourth definition is proposed by Myers [64]. He defines cognition in the glossary as: “All the mental activities associated with thinking, knowing, remembering, and communication.” Although behavior is not defined, there is a definition of “Cognitive learning” as: “The acquisition of mental information, whether by observing events, by watching others, or through language.” No examples are provided of “Non-cognitive learning.” No definition is offered for the word behavior or the behaviorist perspective. Behaviorism is defined as: “The view that psychology (1) should be an objective science that (2) studies behavior without reference to mental processes. Most research psychologists today agree with (1) but not with (2).” Cleary, the second part of this definition is incorrect. There is some information on the behavioral perspective in the introductory chapter but presents only generalizations such as the focus on “how we learn observable responses” ([64], p. 9). 

Schacter, Gilbert, and Wegner [65] offer a fifth definition. Although cognition is not specifically defined, the glossary contains an entry for cognitive psychology. Cognitive psychology is “The scientific study of mental processes, including perception, thought, memory, and reasoning.” Other related entries are: cognitive behavior therapy, cognitive development, cognitive dissonance, cognitive maps, cognitive restructuring, cognitive therapy, and cognitive unconscious. Behaviorism is defined as “An approach that advocates that psychologists restrict themselves to the scientific study of objectively observable behavior.” In the introductory section of the text, Watson and Skinner are discussed. For both individuals only their extreme views are presented. Hull and Spence are mentioned not for their contributions as neobehaviorists but for their views on homeostasis. Edward Tolman is also mentioned in a section on “cognitive elements of operant conditioning.”

A sixth definition is proposed by Wade and Tavris [66]. Although once again there is no definition in the glossary for cognition, they define the cognitive perspective as: “A psychological approach that emphasizes mental processes in perception, memory, language, problem solving, and other areas of behavior.” Other related entries are cognitive dissonance, cognitive schema, and cognitive therapy. It is interesting to note that there is an entry for cognitive ethology, which is defined as “The study of cognitive processes in non-human animals.” Historically, the study of “cognitive processes” is the comparative psychological perspective. Behaviorism is defined as: “An approach to psychology that emphasizes the study of observable behavior and the role of the environment as a determinant of behavior.”

The textbook offered by Wood, Wood and Boyd [67] provides yet another definition—our seventh. Here, cognition is defined in the glossary as: “The mental processes that are involved in acquiring, storing, retrieving, and using information and that includes sensation, perception, imagery, concept formation, reasoning, decision making, problem solving, and language.” Other terms defined are cognitive dissonance, cognitive map, cognitive processes, cognitive therapies, and cognitive therapy. Cognitive psychology is defined as “The school of psychology that sees humans as active participants in their environment; studies mental processes such as memory, problem solving, reasoning, decision making, perception, language, and other forms of cognition.” Behaviorism is defined as “The school of psychology that views observable, measurable behavior as the appropriate subject matter for psychology and emphasizes the key role of environment as a determinant of behavior.” In comparing the definitions of cognitive psychology and behaviorism one gets the impression that behaviorism only study “inactive participants.” 

Our eighth definition of cognition can be found in the Zimbardo, Johnson and McCann [68]. Although not defined in the glossary, the cognitive perspective is defined as “Another of the main psychological viewpoints distinguished by an emphasis on mental processes, such as learning, memory, perception, and thinking, as forms of information processing.” Other cognitive terms in the glossary are cognitive appraisal, cognitive development, cognitive dissonance, cognitive map, cognitive neuroscience, cognitive restructuring, cognitive therapy, and cognitive-behavior therapy. The behavioral perspective is defined as “A psychological viewpoint that finds the source of our actions in environmental stimuli rather than in inner mental processes.” Once again, the referent that behaviorists do not look at “inner mental processes” is wrong. 

I also searched the glossaries and preliminary comments of cognitive texts; the results where the same. I would have expected that in an advanced text the quality and rigor of the definitions would have improved—they did not. Consider the text by Ashcraft and Radvansky [69] who define cognition as “the collection of mental processes and activities used in perceiving, remembering, thinking, and understanding, as well as the act of using those processes.” Reed [70] does not have cognition defined in the glossary but does define cognitive psychology as “The scientific study of cognition.” In the introductory comments cognitive psychology is defined as “The science of how the mind is organized to produce intelligent thought and how it is realized in the brain.” 

One way to estimate the effect that such a variety of definitions have on students is to simply ask them. I asked approximately 70 upper division psychology students to define cognition. The answers were wide ranging and there was no consensus. Representative samples include “The ability to associate and synthesize multiple learned behaviors,” “Mental processes that occur in an organism,” “The ability for an individual to think clearly and have the ability to decipher right from wrong, or myth from reality,” “Mental processes that help solve problems, perform tasks, remember things, and help you function in everyday life,” “The process of thought, attention, memory,” “The process of thinking,” “Internal schemas which include thoughts, feelings, and desires,” “The ability to understand and perform mental abilities and produce constructs,” “Mental processes of the mind through thoughts, feelings, emotions,” “To functionally process thoughts within the mind,” “Mental thought process,” and “The ability to grasp and understand conceptual events.”

## 4. Additional Problems with the Cognitive Perspective not Addressed in Introductory Textbooks

In addition to definitional issues and issues related to the mischaracterization of behaviorism, textbooks fail to inform the student of the many problems associated with cognitive psychology. All that seems to be presented are problems associated with behaviorism as an antiquated perspective incapable of contributing to a “science of the mind.” Consider, for example, that textbooks have little or no discussion of the criteria that makes a process cognitive! One would think that this would be a major issue presented to students—it is not. Students are not told that there are no generally accepted criteria used to decide whether a process is cognitive. Rather they are told that learning, perception, thinking, problem solving, concept formation, *etc*. are examples of cognitive behavior assuming that all such instances must be cognitive. Adams and colleagues have done some excellent work on this issue and propose criteria [3,71]. This work should be included in textbooks and student reading lists.

Overskeid [10] points out further problems with the cognitive perspective. These include, in contrast to popular belief, a narrow research focus, being forced into an almost mystical position on the lack of a physical substrate for mental events, little to no interest in the functional analysis of behavior, and little effort directed toward the study of the influence of motivation and emotion on behavior. The lack of interest in drive on the part of the cognitivists was pointed out over 20 years ago by Amsel [4].

Cromwell and Panksepp [5] echo Amsel’s and Overskeid’s concern about the lack of attention to the motivational and affective in cognitive research. They warn the reader, as others have, that the area of behavioral neuroscience may be in danger by the overuse and misuse of the term cognition. These problems and concerns must be brought to the attention of students if they are to be properly trained in psychology and contribute to psychology as a science. 

James Grice [6,72] points out several serious flaws with respect to data analysis and research designs associated with some aspects of cognitive research. His criticisms continue what now amounts to a chorus of concern of psychological research practices such as failure to encourage replication of results, reliance on group data, scaling issues, and an over-dependence on null hypothesis significance testing. Grice proposes a new method called Observation Oriented Modeling (OOM). OOM has a number of advantages over traditional null hypothesis testing including a reliance on replication, use of distribution free methods, and freedom from estimating abstract population parameters from a sample. Perhaps most importantly, observations are treated as primary and the attributes under investigation are not assumed to be structured as continuous quantities. OOM is now being used in the natural sciences [72] and should find its way into the analysis of cognitive data. 

## 5. Conclusions

This article highlights some of the challenges associated with teaching the behaviorist perspective in the classroom. It is also no easy task. Introductory and cognitive textbook authors must do a better job of incorporating the behaviorist perspective into their texts and bring to the attention of students the many flaws associated with the cognitive perspective. It would also be helpful to correct fundamental errors associated with explaining operant conditioning principles to students [73]. Teachers of psychology must also do a better job of accurately discussing the behaviorist and cognitivist positions.

One way textbook writers can better incorporate the behaviorist position is to have a behaviorist look at the relevant sections. This is also no easy task. All the classical neobehaviorists are deceased and many who call themselves behaviorists have become seduced by the cognitive revolution. Publishers should also have the courage to seek out authors who can write an introductory text, or at the very minimum, contribute supplemental or ancillary materials from the behaviorist perspective. 

A reading list will also be helpful to students. Many of the articles and books cited in this paper can serve as the basis for such a list. Original source material by Watson, Hull, Tolman, Spence, Miller and Skinner should certainly be included. Students will also find fascinating the opinions of Adams, Cromwell, Grice, Overskeid and Panksepp. For professors, I would recommend the little books by Amsel [4] and Lee [7]; these books nicely summarize many of the issues discussed in this paper. 

Texts and reading lists are not enough. Students must be provided hands-on inquiry based activities designed for the behaviorist perspective. One activity I have found especially useful is an application of the scholastic method made popular by Peter Abelard, Thomas Aquinas, and Albertus Magnus. In my version, students are given research articles on a particular topic. The topic is approach from both a behaviorist and cognitivist perspective. Terms are defined and a real attempt is made to identify inconsistencies in definitions, experimental design, data analysis, and interpretation. It is not a debate, but an honest attempt to reconcile the two positions. An excellent example is the work of MacCorquodale and Meehl [54] that compared the work of Hull and Tolman. Another example is the work of Greaves [74] that attempts to find common ground between phenomenology and behaviorism. A third example that I have found useful is for students to consider the use of animals in behavioral research. What do animals have to tell us about human behavior? Muckler [75] is a good article to assign, as is the work of Watson, if the topic of animals in psychology is to be approached scholastically. Before scholastic exercises are attempted, I would encourage the reader to assign a paper on the educational philosophy of Dorothy Sayers [76]. 

Another exercise I have found useful is for students to turn behaviorists into official U.S. Postage stamps. These stamps can include bar codes or QR codes to enable anyone to connect to a website highlighting the individual [77]. They are easy to make and the students enjoy the project. I have used this project in my history of psychology class to make official postage stamps of various behaviorists. 

A further exercise I have found useful is for students to keep a log of instances of conditioning that influence their behavior. This approach uses the form of introspection utilized by Oswald Külpe known as systematic experimental introspection. At first students are surprised that introspection can be used from a behaviorist perspective. As they gain more experience with the technique, many see the advantages in analyzing their own behavior in terms of conditioning principles. Students interpret instances of their behavior in terms of such principles as stimulus intensity, habituation, generalization, history of reinforcement, schedule effects, classical conditioning, *etc*. One way to get them started is to ask students to find instances in literature that can be explained by conditioning principles. Some issues of the *Journal of the Experimental Analysis of Behavior* contain pertinent illustrations and there is a nice example from the dynastic period in Chinese history [78]. 

In addition to the use of scholasticism, stamps, and systematic experimental introspection, students can condition animals in the classroom and be given the opportunity to interpret the results from both the cognitive and behaviorist perspectives. My laboratory has published many papers on conditioning demonstrations suitable for the classroom [79,80]. These demonstrations use inexpensive material and often focus on invertebrate animals. Students quickly see the dangers associated with using cognitive terms to explain the learning of the headless roach, the paramecium, the planarian, the fruit fly, and the honey bee in terms of representations and expectations. Alfred Binet has published a little known book called *The psychic life of micro-organisms: A study in experimental psychology* [81] that is a very interesting read. Frankly, it is alarming to see how the cognitive perspective has infiltrated the invertebrate conditioning literature without any consideration of the various behaviorist positions. 

Avoidance behavior of invertebrates is an excellent example. The key question in avoidance is: how can the absence of an aversive event be reinforcing? The answer is that it must be “expected.” The data obtained with invertebrates suggests that it is not the absence of an expected aversive event that is reinforcing, it is that fact that is paired in a manner readily explained with basic conditioning principles. Consider honey bee avoidance. Bees trained to fly of a target in response to a cue signaling shock will readily learn to do so after a few cue-shock pairings. However, when the bee leaves the target prior to the delivery of the shock, the shock is no longer paired with the cue. Such a situation represents extinction and the bee begins to stay on the target. This is a straightforward application of Pavlovian principles [82]. Moreover, several studies have shown in earthworm and crab that the pairing of a cue with an aversive stimulus gives the same performance as a group of animals that are able to avoid the aversive event by responding to the cue. A cognitive account must predict superior performance in the avoidance groups. Furthermore, a cognitive account of avoidance behavior requires that animals first trained on an avoidance schedule will produce poor performance when their avoidance response no longer is effective (*i.e*., extinction). Rather than produce poor performance the invertebrates continue to respond to the cue [83,84]. In another experiment testing the cognitive interpretation of some aspects of honey bee behavior, it was shown that honey bees will only learn to associate a cue with a feeding when the cue is the presentation of a stimulus. When the cue is the absence of a stimulus, conditioning does not occur [85].

In summary, textbooks authors and faculty must do a better job of presenting the various behaviorist approaches to theory construction. Writers should not fear textbook publishers and journal editors who insist that only “modern” citations (*i.e*., less than 25 years old) be used. Such insistence will further detach the student from a body of literature, and a scientific perspective, that still has much to recommend it—and must be fought. Ancillary materials that are already in the literature should be used to help the student evaluate the behaviorist and cognitive perspectives. Many of us harp on the importance of instilling critical thinking skills in our students yet all of us have run across students and professional researchers that believe cognition can be studied in a snail, tick, planarian, *etc*. without ever defining what cognition is and not presenting criteria on what is and what is not an instance of cognitive behavior. 

Behaviorists also must do a better job of re-asserting our positions not only in the classroom and in print, but also to our colleagues. The behaviorist position is worth fighting for. If not, I fear that as Coffin [20] wrote “So Behaviorism appears as a pathetic figure circling around in the backwash of the widening swiftly flowing stream of science.” ([20], p. 255).
